# Sand Dune Ridge Alignment Effects on Surface BRF over the Libya-4 CEOS Calibration Site

**DOI:** 10.3390/s150203453

**Published:** 2015-02-03

**Authors:** Yves M. Govaerts

**Affiliations:** Rayference, 1030 Brussels, Belgium; E-Mail: yves.govaerts@rayference.eu; Tel.: +32-487-398554

**Keywords:** calibration, radiometry, remote sensing

## Abstract

The Libya-4 desert area, located in the Great Sand Sea, is one of the most important bright desert CEOS pseudo-invariant calibration sites by its size and radiometric stability. This site is intensively used for radiometer drift monitoring, sensor intercalibration and as an absolute calibration reference based on simulated radiances traceable to the SI standard. The Libya-4 morphology is composed of oriented sand dunes shaped by dominant winds. The effects of sand dune spatial organization on the surface bidirectional reflectance factor is analyzed in this paper using Raytran, a 3D radiative transfer model. The topography is characterized with the 30 m resolution ASTER digital elevation model. Four different regions-of-interest sizes, ranging from 10 km up to 100 km, are analyzed. Results show that sand dunes generate more backscattering than forward scattering at the surface. The mean surface reflectance averaged over different viewing and illumination angles is pretty much independent of the size of the selected area, though the standard deviation differs. Sun azimuth position has an effect on the surface reflectance field, which is more pronounced for high Sun zenith angles. Such 3D azimuthal effects should be taken into account to decrease the simulated radiance uncertainty over Libya-4 below 3% for wavelengths larger than 600 nm.

## 1. Introduction

Since the seventies, satellite observations have provided a global view of the Earth, from which it has been possible to generate multi-decadal time series to support global climate monitoring [1]. These first Earth observation satellites were primarily designed for near-real-time meteorological applications with limited requirements on measurement accuracy and precision, at least for short wavelengths. Consequently radiometric calibration, when available, was often limited to pre-launch radiometer characterization. The possibility to generate a reliable climate data record (CDR) from these space observation time series requires, however, tracing them with respect to the radiometric standard. Radiometric calibration necessitates first of all the characterization of a “reference”, defined according to the Système International (SI) standard, against which raw space observations can be compared.

In that context, pseudo-invariant calibration sites (PICSs) play a critical role, because they are suitable for sensor stability monitoring, a prerequisite to CDR generation. Cosnefroy *et al.* [2], who performed early works to identify PICSs, selected a series of about 20 such sites located in the Sahara Desert and Arabian Peninsula. These PICSs were selected according to their spatial uniformity, low cloud cover and precipitation rate. The Committee on Earth Observation Satellites (CEOS) has further refined this list to about six sites for their good spatial and temporal stability. These sites are Mauritania-1, Mauritania-2, Algeria-3, Algeria-5, Libya-1 and Libya-4.

Desert PICSs were originally used for the monitoring of radiometer temporal degradation while in orbit relative to an arbitrarily date. Frank Staylor [3] pioneered using the Libya-4 desert for reflective sensor degradation, collecting 68 months of Advanced Very High Resolution Radiometer (AVHRR) observations. Since then, desert PICSs have been successfully applied for the monitoring of instruments, such as the Landsat Multispectral Scanner, Thematic Mapper, Enhanced Thematic Mapper plus, Moderate Resolution Imaging Spectrometer (MODIS), Visible Infrared Imager Radiometer Suite (VIIRS), *etc.* [4–9]. PICSs are also used for satellite intercalibration with respect to a reference instrument (e.g., [10–14]). Such an approach requires, however, that differences in the spectral characteristics and observation geometries between the compared radiometers are correctly taken into account [15,16]. While extensively applied nowadays, satellite intercalibration cannot be applied on data acquired during the early time of Earth observation, when all available satellite observations were suffering similar radiometric calibration limitations in the shortwave spectral region.

To overcome these limitations, Govaerts and Clerici [17] have developed an approach, where the calibration reference consists of simulated radiances at the satellite level over these bright desert PICSs. The proposed approach, referred to as the simulated calibration reference (SCR), has been successfully applied for the calibration of the VIS band of the Meteosat Visible and Infrared Imager (MVIRI) radiometer on-board the Meteosat First Generation (MFG) series [18]. This continuous dataset starts in 1982, a time when only the AVHRR and Geostationary Operational Environmental Satellite (GOES), both from the National Oceanic and Atmospheric Administration (NOAA, Silver Spring, MD, USA), or Landsat from USA Geological Survey (USGS, Reston, VA, USA) imagers were available. The SCR method requires an accurate characterization of the radiative transfer model parameters influencing the radiance observed at the satellite level. Surface reflectance is described with the so-called Rahman–Pinty–Verstraete (RPV) four-parameter model [19]. This new approach has been widely used since then [20–22]. The estimated SCR mean accuracy, when compared with thousands of well-calibrated observations, is about 3% to 5% [21–23]. A detailed sensitivity analysis has revealed that uncertainties regarding the SCR generation are largely dominated by surface uncertainty characterization, with the exception of the short spectral range, *i.e.*, below 600 nm, where aerosol type and concentration are also contributing to the uncertainties [24].

Among the six desert CEOS PICSs, Libya-4 has proven to be the most stable [25,26], though it has the most complicated topography. This site presents other decisive advantages: its large spatial extension covering an area of about 1° × 1° [2], the absence of vegetation and the existence of numerous datasets collected over Libya-4, as it has already been intensively used as PICSs [27]. This site is composed of long sand dune ridges that might impact the surface bidirectional reflectance factor (BRF) as a function of the Sun azimuth angle. So far, only 1D radiative transfer models (RTMs) have been used for SCR generation. In order to further reduce SCR uncertainties, all possible sources of errors need to be analyzed in detail, especially concerning surface reflectance, as it plays a dominant role above 600 nm. This paper addresses, therefore, the impact on surface BRF of the size of the selected region and the effects of sand dune ridge alignment. Specifically, this work analyzes surface BRF azimuthal dependencies due to sand dune organization for different regions-of-interest (ROIs) sizes using a 3D Monte Carlo ray-tracing RTM [28]. Such an analysis is relevant when observations acquired by mid-morning and mid-afternoon Sun-synchronous polar orbiting satellites are compared.

Section 2 describes the morphology of Libya-4 and identifies various ROIs commonly used. The 3D RTM and experimental modeling setup are described in Section 3. The impact of the ROIs size on BRF is analyzed in Section 4, and the azimuthal dependency is examined in Section 5. This sensitivity analysis showed that Sun azimuth position with respect to sand dune ridge alignment has an effect on the surface reflectance field that is more pronounced for high Sun zenith angles as discussed in Section 6. Such 3D azimuthal effects should be taken into account to decrease the simulated radiance uncertainty over Libya-4 below 3% for wavelengths larger than 600 nm.

## 2. The Libya-4 CEOS Calibration Site

The Libya-4 CEOS calibration site, centered at 28.55° N and 23.39° E in the Great Sand Sea, is composed of spatially-organized sand dunes [29]. Monthly mean precipitation is about 1 mm over that area, with very low cloud cover [2]. The global digital elevation model (DEM) derived from the Advanced Spaceborne Thermal Emission and Reflection Radiometer (ASTER) observations has been used for our sensitivity analysis. This DEM, based on stereo correlation, has a spatial resolution of 1 s or about 30 m at the Equator. The corresponding estimated accuracy, *i.e.*, vertical root-mean-squared-error, ranges between 10 m and 25 m [30].

**Figure 1.**
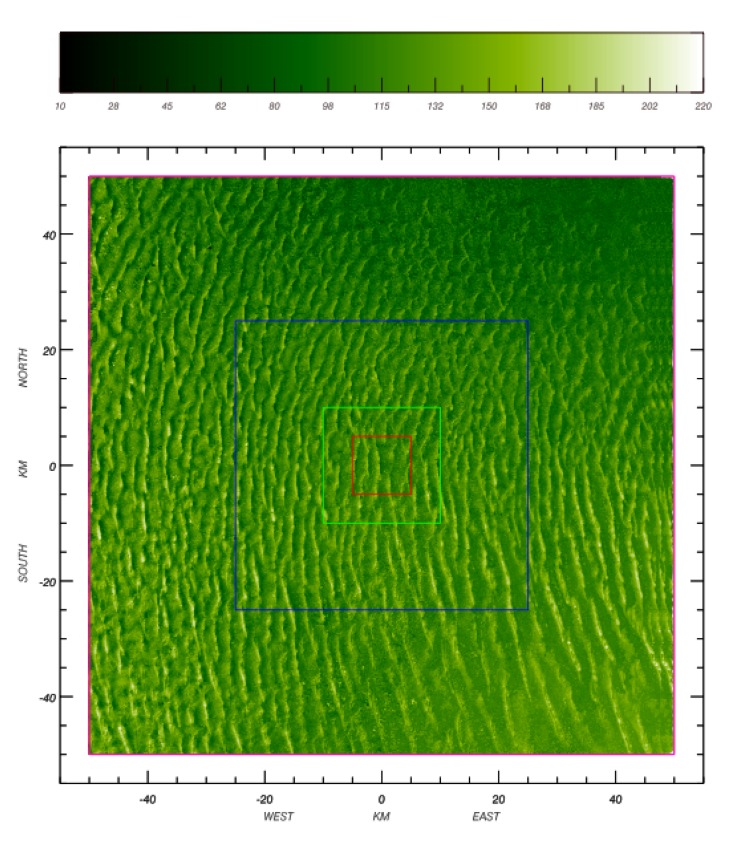
ASTER DEM over the Libya-4 area with the four ROIs used in this study: 10 km × 10 km (red line), 20 km × 20 km (green line), 50 km × 50 km (blue line) and 100 km × 100 km (magenta line). Altitudes are provided in meters.

A visual analysis of the ASTER DEM reveals that the area exhibits a complex multiple-scale spatial organization; Figure 1. The central part of this domain consists of large-scale north-south transverse dunes, *i.e.*, ridges of sand with a steep face in the downwind side [31]. The northeast part of the area has the lowest altitude, populated with the crescent sand dune (barchan) type.

**Figure 2.**
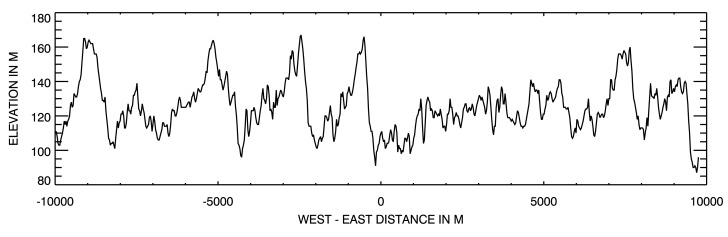
Twenty kilometer-long west-east longitudinal elevation profile over Libya-4 passing through the site center. Distances are given in meters.

Figure 2 shows a 20 km-long longitudinal elevation profile centered at 28.55° N and 23.39° E. Sand dunes are typically 60 m high with an inter-dune distance ranging from 1000 m to 2000 m. The average altitude over that 100 km × 100 km area, shown in Figure 1, is about 120 m, with a standard deviation of 19 m.

Cosnefroy *et al.* [2] originally selected a ROI size of about 100 km × 100 km, or about 1° × 1°, for Libya-4. This original size has been used in many calibration studies (e.g., [11,13,21]). However, other ROI sizes have also been used for Libya-4. Bhatt *et al.* [9] rely on an area of 0.5° × 0.5° to assess the stability of the VIIRS solar channels on-board the Suomi National Polar-orbiting Partnership (S-NPP). An area of about 20 km × 20 km (≈0.2 × 0.2°) is used for the vicarious calibration of the Spinning Enhanced Visible and Infrared Imager (SEVIRI) on-board the Meteosat Second Generation (MSG) [18] and Proba-V [20]. For the purpose of this study, a fourth ROI size of 10 km × 10 km, shown in red in Figure 1, has also been used to assess whether the use of a very small ROI would be possible. An ROI size larger than 100 km is hardly possible, because of the Great Sand Sea border toward the northeast direction. Table 1 shows the elevation characteristics of these four different ROIs. The mean elevation slightly decreases as the ROI size increases. The opposite behavior is observed for the standard deviation.

**Figure 3.**
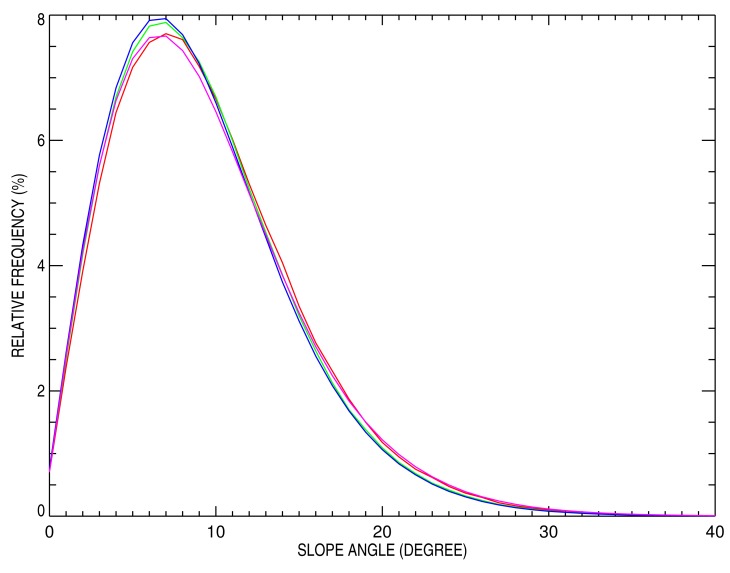
Slope angle relative frequency distribution over the four ROIs: 10 km × 10 km (red line), 20 km × 20 km (green line), 50 km × 50 km (blue line) and 100 km × 100 km (magenta line). Horizontal surfaces have a 0° normal direction.

For each of these ROIs, the slope distribution has been analyzed based on the ATSER DEM. Results are shown in Figure 3. All ROIs exhibit a maximum slope frequency of around 6°–7°. The 20 km × 20 km and 50 km × 50 km ROIs have slightly less slopes with angles higher than 15° compared to the 10 km × 10 km and 100 ×100 km ones. Note that, whatever the ROI size, the maximum sand dune slope or repose angle does not exceed 35°, a number in agreement with expected values for dry sand [32].

**Figure 4.**
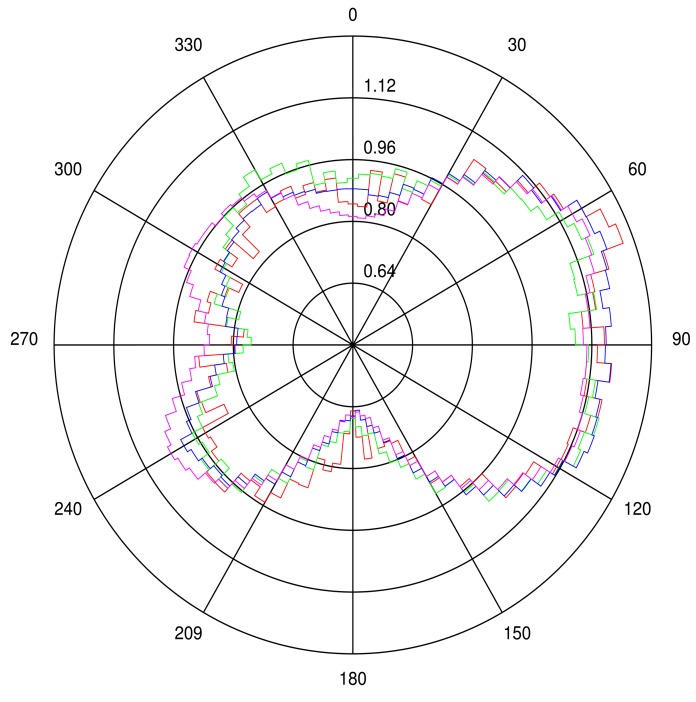
Steep slope, *i.e.*, with a repose angle ranging between 15° and 35°, azimuth direction relative frequency distribution over the four ROIs: 10 km × 10 km (red line), 20 km × 20 km (green line), 50 km × 50 km (blue line) and 100 km × 100 km (magenta line). Circles represent the relative frequency (%) of slope azimuth angles, and polar angles represent the azimuth with zero value pointing to the north.

We next evaluate the azimuthal distribution of the slopes having a repose angles affecting surface BRF, *i.e.*, lying between 15° and 35°; Figure 4. For all ROIs, steep slopes are predominantly pointing toward the east direction, *i.e.*, downwind. The 100 km × 100 km ROI has the most regular slope azimuthal distribution and the smallest one, *i.e.*, 10 km × 10 km, the most irregular one. These steep slopes, observed facing away from the wind direction, cast more shadow than those on the opposite side, resulting in different reflectance magnitude as a function of the illumination azimuth direction. It is therefore expected that this uneven slope azimuth distribution is responsible for BRF effects, depending on the azimuth illumination angle, which will be analyzed in Section 5.

## 3. BRF Simulations

**Figure 5.**
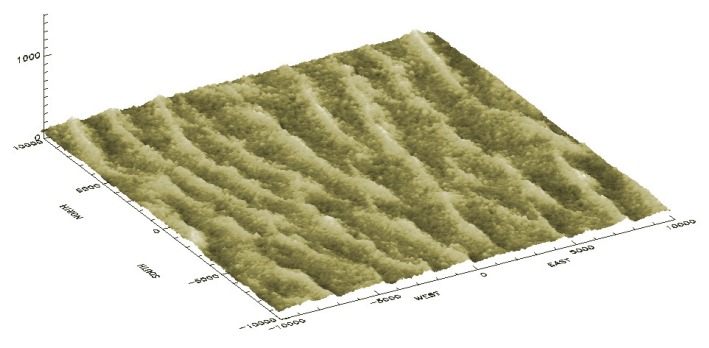
3D scene of Libya-4 based on the ASTER DEM. The 20 × 20 km ROI shown here is centered on 28.55° N and 23.39° W. Distances are given in meters.

Raytran, a three-dimensional (3D) RTM, has been used to analyze the effects of sand dune spatial organization on surface BRF. This RTM allows a 3D scene description of any arbitrary complexity, solving the radiative transfer equation with a Monte Carlo ray-tracing approach [28,33]. This model has been extensively evaluated and has proven to be one of the most accurate surface RTMs [34–38]. It has versatile modeling capabilities, including the simulation of charge-coupled device (CCD) cameras, but also BRF observed at infinity as simulated by 1D RTMs. Within Raytran, the 3D geometrical structure of Libya-4 is constructed with the ASTER DEM, each of the basic geometric elements of the simulated scene being represented by a triangle. As can be seen in Figure 5, there are about 10 large-scale sand dune ridges over the 20 km × 20 km ROI, which corresponds to an inter-ridge distance of about 2 km.

Unfortunately, no Libya-4 in situ sand BRF property measurements are available. More generally, only very limited sand BRF datasets have been acquired. Boucher *et al.* [39] performed some sand BRF measurements at 600 nm and 800 nm in the backscattering direction for a 30° illumination angle. According to these authors, sand BRF is quite Lambertian with a weak backscattering signature. As the primary objective of this paper is to quantify the effects of sand dune spatial organization on surface BRF, we therefore assume a Lambertian sand reflectance. In other words, each triangle representing the sand dune topography within Raytran is assumed to be a Lambertian surface. Consequently, reflectance anisotropy over Libya-4 is only due to topography effects, *i.e.*, sand dune shadows in these Raytran simulations. Secondly, we assume that the sand reflectance properties at a given wavelength are the same over the entire ROI, *i.e.*, all triangles are assigned the same reflectance magnitude. Three different sand reflectance magnitudes have been used for these simulations: 0.15, 0.3 and 0.6. Such values are representative of typical sand reflectance in the visible and near-infrared spectral regions [40].

**Figure 6.**
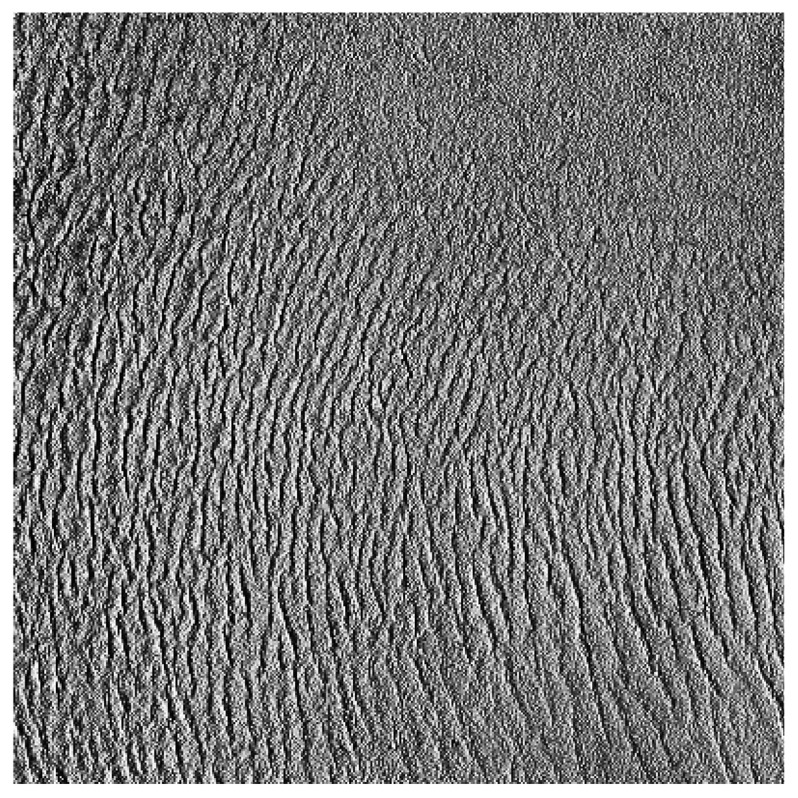
Example of nadir-looking Raytran surface bidirectional reflectance factor (BRF) simulation over Libya-4 for the 100 km × 100 km ROI acquired with a 250 m pixel resolution CCD camera for a sand reflectance value of 0.3. The illumination corresponds to Sun zenith angle (SZA) = 50° and Sun azimuth angle (SAA) = 270°, *i.e.*, from the left side. The mean reflectance of the image is 0.293 with a standard deviation of 0.024. The minimum value is 0.135 and the maximum 0.386.

To clearly quantify the effects of sand dune topography on BRF, all Raytran simulations have been performed at the surface, *i.e.*, without atmospheric scattering effects. All rays are collided into a single illumination direction. An example of a Raytran nadir-looking CCD simulation over the 100 km × 100 km ROI is shown in Figure 6 for a sand surface reflectance of 0.3 and a Sun zenith angle (SZA) of 50°. The Sun azimuth angle (SAA) is equal to 270°. This illumination direction, opposite the steepest slopes, has been selected to emphasis the effect of sand dune shadow on the observed reflectance.

## 4. Effects of Calibration Target Size

In this Section, the effect of Libya-4's various ROI sizes on surface BRF is examined. For that purpose, images with a 250-m pixel resolution, similar to the one shown in Figure 6, have been simulated for both SZA and VZA varying between 0° and 60° and SAA and VAA between 90° and 270°. The objective of these simulations is to determine to what extent the ROI size, characterized by different topographical features, impacts the mean surface BRF value. As seen in Figures 1, 3 and 4, sand dune elevation and alignment slightly differ over the different ROIs. Three different cases have been highlighted each time considering all simulated azimuthal angles: (1) all SZA and VZA angles from 0° to 60°; (2) only high SZA, *i.e.*, larger than 50° and all VZA angles; and (3) only high VZA larger than 50° and all SZA angles. For each of these three cases, the mean BRF and its relative standard deviation have been estimated for the three selected sand reflectance values.

As can be seen in Table 2, the ROI size has no real impact on the mean BRF averaged over all observations and illumination geometries, whatever the angular configuration considered or sand reflectance magnitude, despite the slight differences in the topography characteristics. However, the corresponding relative standard deviation differs notably according to the ROI size. The 50 km side ROI exhibits the smallest standard deviation and the 10 km one the largest. The Libya-4 center area consists of well-organized sand dunes; Figures 1, 2 and 5. Hence, when the ROI size decreases below 50 km, the effects of individual sand dune ridges are exacerbated, increasing thereby the reflectance standard deviation resulting from the contrast between lit and shadow dune faces. Conversely, when the ROI size increases above 50 km, the topography standard deviation increases, as can be seen in Table 1, contributing thus to an increase of the surface BRF standard deviation. It should also be noted that high SZA values have a larger impact on the relative standard deviation than high VZA values. Indeed, high SZA values increase the ratio of cast shadow by sand dunes and, therefore, the BRF spatial variability of the area.

These relative standard deviation values are about twice as large as those reported elsewhere in the literature for the 100 km side ROI. Lacherade *et al.* [11] found that the top-of-atmosphere (TOA) BRF mean relative standard deviation lies between 2.3% and 2.6% over the 100 km × 100 km ROI, and Bouvet [21] reported a value of 3% in all MERIS spectral bands. This difference can be easily explained by the fact that atmospheric scattering processes, which tend to reduce the shadowing effects, are not taken into account in this study. The values have been derived from images acquired at a lower resolution than 250 m.

## 5. BRF SAA Dependency

**Figure 7.**
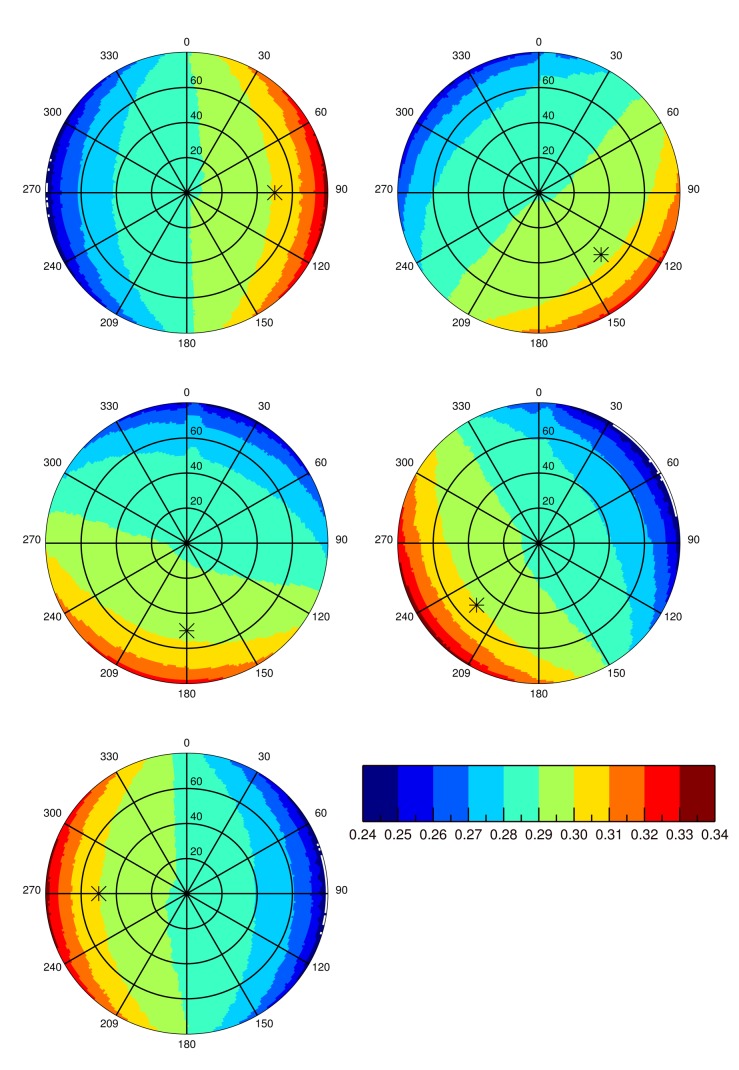
Polar plots of Raytran surface BRF simulations over Libya-4 for the 20 × 20km ROI and SZA = 50°. Sand reflectance is equal to 0.3. Circles represent zenith angles, and polar angles represent azimuth angles with a zero degree azimuth pointing to the north. The * symbol indicates the Sun position.

**Figure 8.**
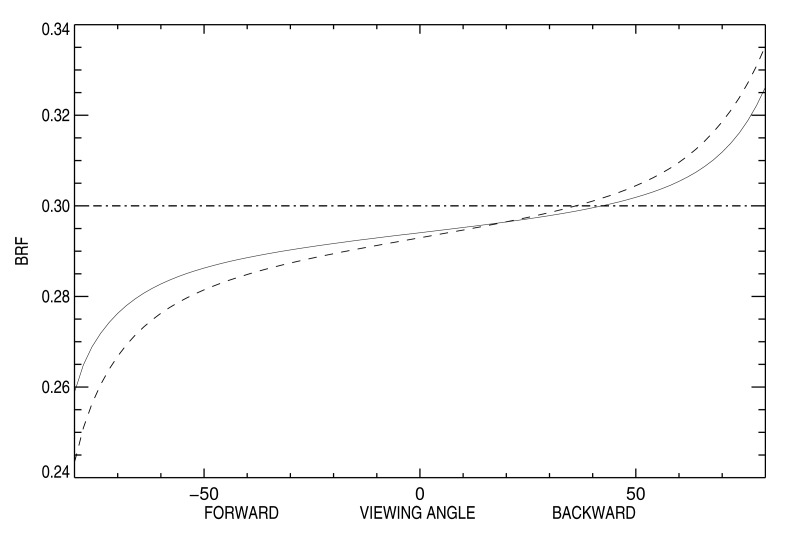
Raytran surface BRF simulation in the principal plane over the 20 × 20 km ROI for SZA = 50°. The solid line corresponds to SAA of 135° and the dashed line to SAA = 225°. The dashed-dotted line shows the sand reflectance level used for the elementary triangles composing the Libya-4 site topography within Raytran.

In the second part of this study, sand dune ridge alignment effects on surface BRF as a function of the SAA are analyzed. Figure 7 shows surface BRF polar plots over the 20 km side ROI for five different SAA values, *i.e.*, 90°, 135°, 180°, 225° and 270°. SZA is set to 50° in this experiment. A first visual inspection of these polar plots reveals the overall backscattering signature resulting from sand dune topography whatever the SAA value. The effects of sand dune ridge alignment on surface BRF are particularly visible when SAA is equal to 180°. In the case of simulations performed with RTM that are only dependent on the actual relative azimuth angle between the Sun and viewing directions, as is the case with the 1D model, the BRF values of the left and right side part of the hemisphere with respect to the principal plane are symmetrical. Such symmetry is clearly not observed in the present case. Additionally, a visual comparison between SAA equal to 135° and 225° plots shows distinct differences between the two illumination conditions. These two SAA configurations correspond to typical mid-morning and mid-afternoon illumination geometry for Sun synchronous polar orbiting radiometers. Figure 4 reveals that the west side of sand dune slopes are steeper than the east side. Consequently, more shadow is cast in the forward direction when SAA equals 225° than when SAA equals 135°, which translates into smaller reflectance values. Conversely, backscattering is more pronounced when SAA equals 225° than 135°. In other words, the BRF departure from a Lambertian surface is more pronounced when sand dune ridges are lit from the west direction than the east one. An analysis of the BRF in the principal plane permits one to better assess this effect. Figure 8 shows the BRF in the principal plane for SZA equal to 50° and SAA set to 135° and 225°. The effects of sand dune morphology are to foster reflectance backscattering, particularly for viewing zenith angles larger than 50°. Conversely, sand dune alignment decreases forward scattering, notably for viewing zenith angles larger than 50°. This behavior is observed independently of the sand reflectance magnitude.

**Figure 9.**
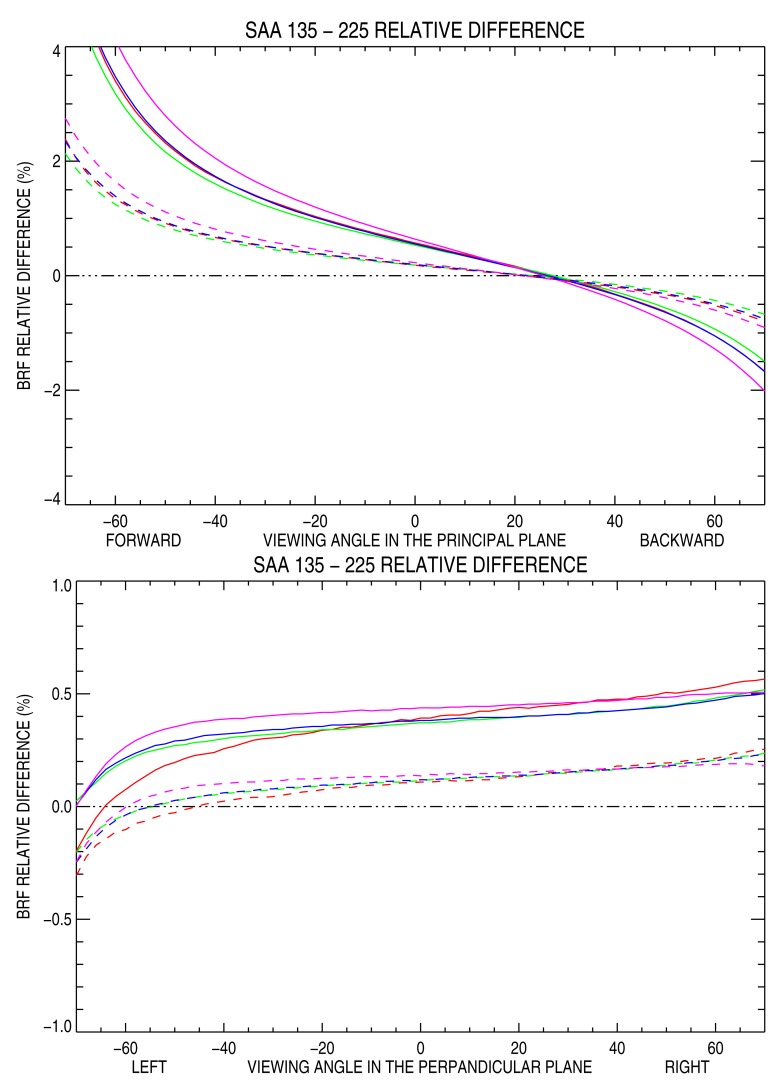
BRF relative difference between SAA 135°–225° in the principal plane (**top**) and the perpendicular one (**bottom**). Solid lines are for SZA = 50° and dashed lines are for SZA = 25° over the four ROIs: 10 × 10 km (red line), 20 × 20 km (green line), 50 × 50 km (blue line) and 100 × 100 km (magenta line). Sand reflectance is set to 0.3.

To further quantify the difference between these two observation conditions, the surface BRF relative difference between SAA equal to 135° and 225° has been estimated in the principal and perpendicular planes over the four different ROIs. Results are shown in Figure 9 for two different SZA values, *i.e.*, 25° and 50°. In the principal plane, relative differences are more pronounced in the forward than in the backscattering direction. The relative differences are in the range -2%–+4% when SZA is equal to 50° and -1%–+2% when SZA equals 25°. These differences are twice as small when VZA is restricted to the [-40°, +40°] interval. The largest relative difference occurs on the 100 km × 100 km ROI, while the other three ROIs have a very similar behavior. These findings are in good agreement with the results of Table 2, showing that the ROI size has limited impact on the surface BRF mean value.

In the perpendicular plane, differences are almost always positive, *i.e.*, the SAA equal to 135° case has larger values as a result of less pronounced forward scattering from that azimuthal direction than from the 225° one. Additionally, a minor asymmetry is observed between the left and right side of the perpendicular plane, particularly for the 10 km side ROI for VZA values larger than 40°.

## 6. Discussion and Conclusions

Desert PICSs, such as Libya-4, were originally used for sensor stability monitoring relative to a given date and, more recently, for radiometer intercalibration. This latter approach requires spectral band adjustment in case the spectral characteristics of the compared instruments are not equal and is often limited to simultaneous nadir overpass. To overcome these limitations, simulated TOA radiances or reflectances over bright desert PICSs have been proposed as the calibration reference, relying either on empirical (e.g., [22,41]) or physically-based simulations (e.g., [21,23]). The former approach is pretty simple to develop and often depends on instrument observations used to fit the empirical model parameters. It should therefore be considered as a radiometer intercomparison method. Additionally, interpolation or extrapolation outside the fitted interval might be hazardous. On the other hand, physically-based models, though more complicated to elaborate, present several distinctive advantages. They can better account for the molecular absorption features in the atmosphere and are more reliable when extrapolated outside the data range that has been used to “tune” the surface parameters.

Currently, the SCR approach has proven to be accurate within 3% to 5% when several years, *i.e.*, seasonal cycles, of observations are processed. Sensitivity analyzes have shown that, over bright surfaces, TOA BRF is essentially determined by the magnitude of surface reflectance with the exception of the short wavelength spectral region, *i.e.*, below 600 nm, where atmospheric scattering processes equally contribute to the TOA BRF magnitude [24]. Hence, an accurate characterization of surface BRF is critical to further reduce SCR uncertainty, thereby improving CDR generation reliability [42]. Such an effort could be compared to the one that has been performed to characterize the Spectralon® panel BRF used for on-board calibration [43]. This study aims at decreasing PICS SCR uncertainty below the 3% value currently reached.

This work focuses on the surface BRF characterization of Libya-4, one of the most stable CEOS PICSs. The effects of sand dune ridge alignments and ROI size on surface BRF are specifically analyzed with a 3D RTM. The scene construction relies on the 30-m resolution ASTER DEM. Four different ROI sizes, varying between 10 km to 100 km, all sharing the same central position, are analyzed.

Within the framework of the study assumptions, it has been shown that ROI size has a pretty limited impact on the mean BRF averaged over a large number of illumination and viewing conditions. The choice of the ROI size is therefore not critical. However, the 10 km side ROI exhibits the largest surface BRF standard deviation, and it is therefore preferable not to use such a small ROI. On the other hand, a too large of an ROI might not be the best choice, too. It increases the probability of cloud contaminations. Additionally, VZA variations within the 100 km size ROI might introduce undesirable effects due to different pathlengths in the atmosphere. The 50 km side ROI shows the smallest surface BRF standard deviation and is thus recommended for data acquisition over Libya-4.

It has been shown that Sun azimuth angle has a more pronounced effect on surface BRF than the ROI size. Two illumination azimuth angles of, respectively, 135° and 225° have been analyzed in detail. They correspond to typical mid-morning and mid-afternoon Sun synchronous polar orbiting radiometer acquisition conditions. The principal plane surface BRF difference between these two illumination directions can exceed 1%, especially when the SZA is large. In the perpendicular plane, the difference does not exceed 0.5%.

Various aspects of this work might require further developments. Firstly, it is assumed that sand reflectance is Lambertian. A non-Lambertian sand reflectance, while worthwhile to apply for the simulation of TOA BRF, would not significantly affect sand dune shadowing effects on the reflectance, and the 3D effects would essentially remain the same. Secondly, the study also assumes uniform sand reflectance over the Libya-4 area. Unfortunately, no *in situ* observations of sand properties are available. However, it should be possible to deduce the importance of some of these properties from the literature. Sand reflectance is essentially determined by grain size, shape and chemical composition [44]. Absorption is primarily controlled by the concentration of iron oxide [40]. No information is available concerning changes in the sand chemical composition over that area. Sand reflectance depends also on grain size. A small effective radius increases the reflectance [45]. According to these authors, a decrease of the effective radius from 500 *μ*m to 50 *μ*m leads to a 4% reflectance increase at 1000 nm. Sand grain size and composition distribution act as important factors in the morphology of wind-blown sand landforms and the dynamic processes of their formation [46]. These authors found that the contents of clay and silt are highest on interdune areas, lowest on the crests and higher on the leeward slopes than on the windward slopes. The contents of very fine and fine grains are highest on the windward slopes and lowest on the crests. Hence, such information, combined with the sand reflectance model proposed by [47], could be used to simulate the spatial heterogeneity of Libya-4 sand reflectance realistically. Finally, atmospheric effects have not been taken into account in this study. At a short wavelength, *i.e.*, below 600 nm, the surface BRF effects analyzed in this paper are attenuated by atmospheric scattering processes. At longer wavelengths, the only significant atmospheric processes are molecular absorption, so that the conclusions of this study apply also to TOA BRF and should be taken into account to decrease SCR uncertainty below 3%.

## Figures and Tables

**Table 1.** Statistical elevation values over the 10 km × 10 km, 20 km × 20 km, 50 km × 50 km and 100 km × 100 km ROIs.

**ROI Size (km)**	**Min (m)**	**Max (m)**	**Mean (m)**	**SD (m)**
10	73	184	124	14
20	68	208	123	14
50	56	215	122	16
100	11	222	120	19

**Table 2.** Effect of Libya-4 ROI size on mean surface BRF and the relative standard deviation (SD).

	**All Angles**		**SZA** > **50°**		**VZA**> **50°**	

**ROI Size (km)**	**Mean BRF**	**Rel. SD** (%**)**	**Mean BRF**	**Rel. SD**	**Mean BRF**	**Rel. SD** (%**)**
Sand Reflectance = 0.150						

10	0.147	6.76	0.147	8.53	0.148	8.17
20	0.147	5.26	0.147	7.30	0.147	5.52
50	0.147	5.05	0.147	7.17	0.148	5.14
100	0.147	5.76	0.147	7.90	0.147	5.44

Sand Reflectance = 0.300						

10	0.295	6.74	0.294	8.51	0.296	8.16
20	0.295	5.24	0.295	7.28	0.296	5.50
50	0.295	5.03	0.295	7.15	0.296	5.13
100	0.295	5.74	0.294	7.88	0.296	5.42

SandRreflectance = 0.600						

10	0.593	6.70	0.591	8.48	0.595	8.13
20	0.593	5.21	0.592	7.25	0.595	5.48
50	0.593	5.00	0.593	7.13	0.595	5.12
100	0.593	5.71	0.592	7.84	0.595	5.39

## Abbreviations

ASTER: Advanced Spaceborne Thermal Emission and Reflection Radiometer
AVHRR: Advanced Very High Resolution Radiometer
BRF: bidirectional reflectance factor
CCD: charge-coupled device
CDR: climate data record
CEOS: Committee on Earth Observation Satellites
DEM: digital elevation model
GOES: Geostationary Operational Environmental Satellite
MSG: Meteosat Second Generation
MFG: Meteosat First Generation
MODIS: Moderate Resolution Imaging Spectrometer
MVIRI: Meteosat Visible and Infrared Imager
NOAA: National Oceanic and Atmospheric Administration
PICS: pseudo-invariant calibration sites
ROI: region-of-interest
RTM: radiative transfer model
RPV: Rahman–Pinty–Verstraete
SAA: Sun azimuth angle
SCR: simulated calibration reference
SD: standard deviation
SEVIRI: Spinning Enhanced Visible and Infrared Imager
SI: Système International
SZA: Sun zenith angle
S-NPP: Suomi National Polar-orbiting Partnership
USGS: U.S. Geological Survey
VAA: viewing azimuth angle
VIIRS: Visible Infrared Imager Radiometer Suite
VZA: viewing zenith angle
